# Revisiting Older Drivers’ Risks of At-fault Motor Vehicle Collisions in Japan

**DOI:** 10.2188/jea.JE20230217

**Published:** 2024-06-05

**Authors:** Masao Ichikawa, Haruhiko Inada, Shinji Nakahara

**Affiliations:** 1 Department of Global Public Health, Institute of Medicine, University of Tsukuba, Ibaraki, Japan; 2 Department of Public Health and Health Policy, Graduate School of Medicine, The University of Tokyo, Tokyo, Japan; 3 Graduate School of Health Innovation, Kanagawa University of Human Services, Kanagawa, Japan

**Keywords:** aging, drivers, traffic crashes, traffic injuries

## Abstract

**Background:**

In Japan, older drivers have been encouraged to surrender their driving licenses for traffic safety, despite the potential adverse social and health outcomes of driving cessation. We reconsidered such policies and social pressure by comparing the risk of at-fault motor vehicle collisions (MVCs) across the age groups of drivers.

**Methods:**

Using the national data of police-reported MVCs that occurred between 2016 and 2020, we examined the number of at-fault MVCs per licensed driver (MVC rate) and the number of fatally and non-fatally injured persons per at-fault MVC by the sex and age groups of at-fault drivers.

**Results:**

The MVC rate of older drivers was higher than that of middle-aged drivers but lower than that of young drivers. The number of injured persons among the collided counterparts (collided car occupants, motorcyclists, bicyclists, and pedestrians) per MVC caused by older drivers was not greater than that by drivers in other age groups. In fatal MVCs caused by older drivers, drivers themselves or their passengers tend to be killed rather than their collided counterparts. Overall, the results were mostly consistent between male and female drivers.

**Conclusion:**

The risk of at-fault MVCs increased with the advancing age of drivers after middle age; however, this risk among older drivers did not exceed that among young drivers, without posing a high risk of injuries to their collided counterparts.

## INTRODUCTION

The driver population is getting older with population aging. As the number of older drivers increases, the proportion of their motor vehicle collisions (MVCs) also increases. In Japan, which has the oldest population in the world, people aged ≥65 years accounted for 29% of the total population and 24% of the driver population in 2021.^1^^,^^2^ From 2012 to 2021, the proportion of MVCs caused by drivers aged ≥65 years rose from 16% to 24%.^3^ Consequently, their MVCs, especially fatal ones, have gained media attention, and public concern has been raised regarding the safety of older drivers.

Stringent licensing policies are imposed on older drivers because their driving abilities could be affected by functioning difficulties as they age. However, it is reported that older drivers adjust their driving by driving less or avoiding challenging situations, compensating for their declining abilities^4^^–^^6^ and potentially attenuating their MVC risk. Nevertheless, older drivers in Japan are not only required to take driving lessons and cognitive screening tests at their license renewal but are further encouraged to surrender their driving licenses.^7^

A policy that promotes older drivers’ surrendering of their driving licenses will reduce the number of MVCs; however, unless alternative transportation is available, driving cessation will restrict mobility, which might induce adverse effects on physical, social, and mental well-being.^8^^–^^11^ This is a critical issue in car-dependent societies, such as Japan, where 57% of people aged ≥60 years drive cars regularly.^12^ Additionally, it should be noted that driving cessation turns drivers to vulnerable road users, such as pedestrians and bicyclists. In Japan, the proportion of vulnerable road users in road traffic deaths is over 50%.^13^ This is another concern over older drivers’ surrendering of their driving licenses.

Older drivers’ risk of injuring others is not as high as the media touts in Japan.^14^^–^^21^ Policy decisions should be based on a comparison between the risk of injury to other road users by older drivers and the risk of adverse events to older people with limited mobility. We previously examined the former risk, but our previous study had the limitations of the short temporal and limited geographical coverage of MVCs and used data from more than 10 years ago.^14^ Therefore, in the present study, we used recent nationwide and long-term data to examine the crash and injury profiles of MVCs caused by older drivers and drivers in other age groups to reconsider traffic safety policies and social pressure to promote driving cessation among older drivers.

## METHODS

### Data

In this descriptive cross-sectional study, we obtained data on at-fault MVCs from the Institute for Traffic Accident Research and Data Analysis, which compiles police-reported MVC data from the National Police Agency. The data included the number of at-fault MVCs by collided counterparts (cars; motorcycles, including mopeds; bicycles; pedestrians; others, such as trains; and none [single crashes]), and the number of deaths, serious injuries, and minor injuries by at-fault drivers, their passengers, and collided counterparts. The data covered MVCs that occurred between January 2016 and December 2020 and were stratified by the sex and age group of at-fault drivers (18–19 years, 5-year intervals from 20 to 84 years, and ≥85 years). Only MVCs in which at-fault drivers drove cars were included because this was the interest of this study. We excluded at-fault MVCs collided with “others” from the analysis because the numbers of such MVCs caused by male and female drivers were as small as 373 and 158, respectively, for the entire study period. We obtained the number of licensed drivers by sex and age group from the driving license statistics of the National Police Agency.^22^^–^^26^

In the police-reported MVC data, deaths are defined as deaths occurring within 24 hours of the crash, serious injuries as injuries diagnosed by physicians requiring treatment for at least 30 days, and otherwise minor injuries.^27^ The driver’s responsibility for collisions was determined through police investigations at the scene of crashes.^27^ Drivers determined to be primarily responsible for crashes, including single-vehicle crashes, were referred to as at-fault drivers.

### Analysis

First, we calculated the number of at-fault MVCs per 100,000 licensed drivers per year (at-fault MVC rate) by collided counterparts according to the sex and age group of at-fault drivers, to examine the extent to which older drivers caused MVCs by their collided counterparts. Next, we calculated the number of injured persons (all injuries [fatal, serious, or minor injuries]), and fatally or seriously injured persons per 100 at-fault MVCs among at-fault drivers, their passengers, collided car occupants, motorcyclists, bicyclists, and pedestrians by the sex and age group of at-fault drivers, to examine how serious, on average, the consequences of MVCs caused by older drivers were. Finally, we calculated the proportion of at-fault drivers, their passengers, collided car occupants, motorcyclists, bicyclists, and pedestrians among all deaths in fatal MVCs by the sex and age group of at-fault drivers, to examine who was killed in fatal MVCs caused by older drivers.

## RESULTS

During the 5-year study period, 1,888,652 at-fault MVCs (467 per 100,000 licensed drivers per year) occurred, including 1,138,063 car-to-car crashes (60%), 208,783 car-to-motorcycle crashes (11%), 312,252 car-to-bicycle crashes (17%), 195,048 car-to-pedestrian crashes (10%), and 34,506 single crashes (2%).

The at-fault MVC rate was higher among males than among females, irrespective of their age (Table 1). Among both sexes, the at-fault MVC rate tended to increase as the age of the at-fault drivers advanced after middle age. However, among males, the at-fault MVC rate was the highest among drivers aged 18–19 years (1,811), followed by drivers aged 20–24 years (1,034) and 25–29 years (762), whereas it was 499, 548, 595, and 661 among drivers aged 70–74, 75–79, 80–84, and ≥85 years, respectively. Among females, the at-fault MVC rate was the highest among drivers aged 18–19 years (964), and it was almost the same among drivers aged 20–24 years (572) and drivers aged ≥85 years (571), followed by drivers aged 80–84 years (530). Regarding collided counterparts, the at-fault MVC rate with cars was higher than that with other counterparts in all age groups of at-fault drivers for both sexes.

**Table 1.** Number of licensed drivers and their at-fault motor vehicle crashes (MVCs) and the at-fault MVC rate (per 100,000 licensed drivers per year) by collided counterparts and the sex and age group of at-fault drivers from 2016 to 2020

Driver sex	Driver age, years	Licensed drivers^a^	At-fault MVCs^a^	At-fault MVC rate	At-fault MVC rate by collided counterparts				

					Cars	Motorcycles	Bicycles	Pedestrians	None (Single crashes)
Male	18–19	2,107,092	38,155	1,811	1,437	130	113	71	60
	20–24	12,470,745	128,983	1,034	773	95	93	54	19
	25–29	14,340,498	109,302	762	532	79	89	52	10
	30–34	16,538,253	99,525	602	395	68	83	47	8
	35–39	18,751,691	97,849	522	326	62	79	47	7
	40–44	21,984,599	111,270	506	302	62	84	51	7
	45–49	23,473,178	117,553	501	281	64	91	56	8
	50–54	20,179,691	100,262	497	271	62	95	59	9
	55–59	18,293,562	91,164	498	269	60	97	62	11
	60–64	17,643,035	91,243	517	270	62	106	68	12
	65–69	20,122,970	103,468	514	260	63	107	71	13
	70–74	16,685,782	83,263	499	253	58	105	70	13
	75–79	11,021,417	60,389	548	296	57	108	71	15
	80–84	6,071,805	36,146	595	345	52	103	74	21
	≥85	2,538,004	16,785	661	411	48	97	71	34

	Total	222,222,322	1,285,357	578	347	65	95	60	12

Female	18–19	1,652,909	15,937	964	799	54	53	36	23
	20–24	10,628,589	60,808	572	442	44	50	27	8
	25–29	12,669,259	49,768	393	275	37	50	27	5
	30–34	14,892,213	46,396	312	199	34	49	26	3
	35–39	17,182,465	51,199	298	179	35	53	29	3
	40–44	20,258,171	62,127	307	176	37	58	33	2
	45–49	21,639,402	63,711	294	162	37	58	35	2
	50–54	18,449,686	50,085	271	147	33	55	33	3
	55–59	16,391,420	43,285	264	144	30	53	33	4
	60–64	15,115,863	40,900	271	149	30	52	35	4
	65–69	15,575,265	47,230	303	167	31	58	41	6
	70–74	10,646,893	36,955	347	194	34	64	47	9
	75–79	4,991,545	23,156	464	264	40	83	61	16
	80–84	1,792,733	9,506	530	312	40	84	68	26
	≥85	391,203	2,232	571	346	38	87	67	32

	Total	182,277,616	603,295	331	201	35	56	34	5

^a^Sum of the number of licensed drivers or at-fault MVCs in each year from 2016 to 2020.

Across the age groups of at-fault drivers, the at-fault MVC rate with cars and motorcycles was much higher among young drivers than among other drivers, especially among males. However, the at-fault MVC rate with bicycles and pedestrians was relatively higher among older drivers than among other drivers. The single crash rate was high toward both the upper and lower ends of the age groups of at-fault drivers.

The number of injured persons per 100 MVCs was much higher among collided car occupants (73.5 and 73.0 per 100 MVCs caused by male and female drivers, respectively) than bicyclists (16.6 and 17.0), motorcyclists (11.4 and 10.6), pedestrians (10.3 and 10.4), at-fault drivers (3.3 and 5.7), and at-fault drivers’ passengers (2.7 and 1.9) (Table 2). Across the age groups of at-fault drivers, the number of injured persons among collided car occupants and motorcyclists in MVCs caused by older drivers was mostly lower than the average, whereas the number of injured persons among at-fault drivers, passengers, bicyclists, and pedestrians in MVCs caused by older drivers was mostly higher than the average.

**Table 2.** Number of injured persons (all injuries [fatal, serious, or minor injuries] and fatal or serious injuries) per 100 at-fault motor vehicle crashes among at-fault drivers, their passengers, collided car occupants, motorcyclists, bicyclists, and pedestrians by the sex and age group of at-fault drivers from 2016 to 2020

Driver sex	Driver age, years	At-fault drivers		At-fault drivers’ passengers		Collided car occupants		Motorcyclists		Bicyclists		Pedestrians	
					
		All	Fatal/ serious	All	Fatal/ serious	All	Fatal/ serious	All	Fatal/ serious	All	Fatal/ serious	All	Fatal/ serious
Male	18–19	5.3	0.9	6.3	1.5	98.6	2.3	7.3	1.2	6.3	0.7	3.9	1.0
	20–24	3.9	0.6	3.1	0.6	92.4	1.8	9.3	1.4	9.1	1.0	5.2	1.2
	25–29	3.5	0.5	2.1	0.3	86.1	1.5	10.5	1.5	11.8	1.1	6.8	1.5
	30–34	3.4	0.5	1.9	0.3	80.9	1.4	11.5	1.6	14.0	1.3	7.9	1.7
	35–39	3.1	0.5	1.9	0.3	76.9	1.4	12.0	1.6	15.3	1.4	9.0	2.0
	40–44	2.9	0.5	1.8	0.3	73.3	1.3	12.5	1.7	16.7	1.5	10.1	2.2
	45–49	2.6	0.6	2.0	0.2	69.2	1.2	12.9	1.8	18.4	1.6	11.3	2.4
	50–54	2.8	0.6	2.2	0.3	67.0	1.3	12.6	1.7	19.3	1.6	11.9	2.4
	55–59	2.7	0.7	2.9	0.4	65.7	1.3	12.1	1.7	19.7	1.6	12.4	2.5
	60–64	2.8	0.8	3.0	0.4	63.4	1.3	12.1	1.7	20.7	1.6	13.1	2.6
	65–69	3.1	1.0	3.6	0.5	61.2	1.3	12.4	1.8	21.0	1.7	13.8	2.5
	70–74	3.7	1.3	3.4	0.6	61.0	1.4	11.7	1.6	21.3	1.7	14.0	2.5
	75–79	4.7	1.6	2.9	0.8	64.7	1.5	10.6	1.7	20.0	1.7	13.0	2.3
	80–84	6.4	2.7	3.0	1.1	68.9	1.6	8.8	1.4	17.6	1.8	12.4	2.7
	≥85	8.9	4.2	3.4	1.5	73.4	1.7	7.4	1.3	14.8	1.8	10.7	2.5

	Total	3.4	0.8	2.7	0.5	73.5	1.4	11.4	1.6	16.6	1.4	10.3	2.1

Female	18–19	7.2	0.7	3.7	0.7	101.8	2.0	5.6	1.0	5.5	0.7	3.7	0.9
	20–24	6.0	0.5	2.1	0.3	93.2	1.4	7.7	1.2	8.9	0.9	4.8	1.1
	25–29	5.8	0.4	1.9	0.2	84.2	1.3	9.5	1.4	12.9	1.1	6.8	1.5
	30–34	5.5	0.3	2.0	0.2	77.0	1.1	10.9	1.4	16.0	1.2	8.4	1.7
	35–39	5.2	0.3	2.1	0.2	72.1	1.1	11.7	1.7	18.1	1.5	9.6	1.8
	40–44	4.5	0.3	1.7	0.2	69.4	1.0	12.3	1.8	19.1	1.4	10.8	2.0
	45–49	4.4	0.4	1.5	0.2	66.5	1.1	12.8	1.7	20.0	1.5	11.7	2.2
	50–54	4.9	0.5	1.5	0.2	65.5	1.1	12.3	1.7	20.4	1.5	12.3	2.3
	55–59	5.4	0.8	1.6	0.3	65.9	1.2	11.4	1.7	20.2	1.5	12.6	2.4
	60–64	5.6	1.0	1.5	0.4	66.1	1.4	11.2	1.6	19.4	1.7	13.0	2.6
	65–69	6.2	1.1	1.7	0.4	65.8	1.4	10.4	1.6	19.2	1.8	13.5	2.7
	70–74	6.9	1.6	1.9	0.5	66.8	1.5	9.8	1.5	18.5	1.7	13.6	2.7
	75–79	8.1	2.0	2.1	0.7	67.0	1.4	8.7	1.5	18.0	1.7	13.1	2.9
	80–84	10.4	3.3	2.6	1.0	68.4	1.5	7.6	1.2	15.9	1.6	12.9	2.7
	≥85	11.9	4.3	1.7	0.5	68.9	2.3	6.9	1.3	15.5	1.7	11.8	3.0

	Total	5.7	0.7	1.9	0.3	73.0	1.3	10.6	1.5	17.0	1.4	10.4	2.1

The number of fatally or seriously injured persons per 100 MVCs was the highest among pedestrians (2.1 and 2.1 per 100 MVCs caused by male and female drivers, respectively), followed by motorcyclists (1.6 and 1.5), bicyclists (1.4 and 1.4), collided car occupants (1.4 and 1.3), at-fault drivers (0.8 and 0.7), and at-fault drivers’ passengers (0.5 and 0.3). Typically, there were no distinct differences in the number of fatally or seriously injured persons per MVC among collided car occupants and motorcyclists irrespective of the age group and sex of at-fault drivers, and the number of fatally or seriously injured persons among bicyclists and pedestrians in MVCs caused by older drivers was almost equivalent to that in MVCs caused by middle-aged drivers. However, the number of fatally or seriously injured persons among at-fault drivers and their passengers in MVCs caused by older drivers tended to be greater as the drivers advanced in age.

The proportion of at-fault driver deaths among all deaths in fatal MVCs caused by older drivers was much higher than that in fatal MVCs caused by other drivers; older drivers tended to be killed in their fatal MVCs (Table 3). This proportion was 31% in MVCs caused by all male drivers, whereas it was 43%, 53%, 60%, and 67% in MVCs caused by male drivers aged 70–74, 75–79, 80–84, and ≥85 years, respectively. The proportion of at-fault drivers’ passenger deaths among all deaths was higher in MVCs caused by young and older drivers than in those caused by other drivers, especially among males. In contrast, the proportion of pedestrian deaths among all deaths in fatal MVCs caused by older drivers was smaller than that in fatal MVCs caused by other drivers, as was the proportion of deaths of collided car occupants, motorcyclists, and bicyclists among all deaths in fatal MVCs caused by older drivers. The proportion of pedestrian deaths among all deaths was 38% in fatal MVCs caused by all male drivers, whereas it was 32%, 24%, 14%, and 11% in fatal MVCs caused by male drivers aged 70–74, 75–79, 80–84, and ≥85 years, respectively. Generally, similar trends were observed among female drivers.

**Table 3.** Number and proportion of at-fault drivers, their passengers, collided car occupants, motorcyclists, bicyclists, and pedestrians among all deaths in fatal motor vehicle crashes by the sex and age group of at-fault drivers from 2016 to 2020

Driver sex	Driver age, years	At-fault drivers		At-fault drivers’ passengers		Collided car occupants		Motorcyclists		Bicyclists		Pedestrians	
Male	18–19	67	25%	65	24%	30	11%	27	10%	14	5%	63	24%
	20–24	195	25%	93	12%	71	9%	83	11%	68	9%	261	34%
	25–29	123	19%	60	9%	62	10%	62	10%	57	9%	282	44%
	30–34	126	20%	24	4%	41	6%	55	9%	76	12%	309	49%
	35–39	132	20%	23	4%	44	7%	49	7%	77	12%	331	50%
	40–44	168	20%	35	4%	66	8%	99	12%	91	11%	399	47%
	45–49	199	21%	23	2%	60	6%	107	11%	125	13%	455	47%
	50–54	207	24%	35	4%	68	8%	64	7%	115	13%	369	43%
	55–59	219	28%	36	5%	50	6%	56	7%	94	12%	325	42%
	60–64	226	30%	42	6%	49	6%	46	6%	71	9%	324	43%
	65–69	315	37%	82	10%	35	4%	63	7%	56	7%	293	35%
	70–74	329	43%	66	9%	31	4%	41	5%	47	6%	247	32%
	75–79	337	53%	83	13%	11	2%	26	4%	31	5%	150	24%
	80–84	336	60%	78	14%	14	3%	19	3%	34	6%	79	14%
	≥85	238	67%	55	15%	6	2%	5	1%	13	4%	39	11%

	Total	3,217	31%	800	8%	638	6%	802	8%	969	9%	3,926	38%

Female	18–19	11	19%	12	21%	12	21%	6	11%	3	5%	13	23%
	20–24	36	20%	19	11%	9	5%	16	9%	16	9%	83	46%
	25–29	16	10%	15	9%	12	7%	21	13%	17	10%	82	50%
	30–34	20	12%	12	7%	10	6%	18	11%	16	10%	91	54%
	35–39	19	11%	23	14%	7	4%	25	15%	15	9%	77	46%
	40–44	22	10%	17	7%	7	3%	37	16%	22	10%	125	54%
	45–49	32	13%	13	5%	6	2%	32	13%	21	9%	139	57%
	50–54	28	14%	21	10%	18	9%	26	13%	18	9%	94	46%
	55–59	28	14%	30	15%	10	5%	24	12%	16	8%	98	48%
	60–64	47	21%	35	16%	6	3%	19	9%	16	7%	99	45%
	65–69	68	25%	22	8%	9	3%	23	8%	25	9%	125	46%
	70–74	82	36%	24	11%	8	4%	14	6%	14	6%	83	37%
	75–79	95	48%	25	13%	2	1%	10	5%	9	5%	59	30%
	80–84	59	51%	13	11%	2	2%	10	9%	7	6%	25	22%
	≥85	20	67%	2	7%	1	3%	2	7%	1	3%	4	13%

	Total	583	22%	283	11%	119	4%	283	11%	216	8%	1,197	45%

## DISCUSSION

The at-fault MVC rate increased with the advancing age of drivers after middle age, but the rate among older drivers was still lower than that among young drivers. Moreover, the total number of injured persons per MVC caused by older drivers was not excessively higher than that caused by drivers in other age groups. In the fatal MVCs of older drivers, the drivers themselves and their passengers tended to be killed more than their counterparts. This was not the case for drivers in other age groups. Overall, the results were mostly consistent between male and female drivers.

It should be noted that older drivers cause more collisions with bicyclists and pedestrians than other drivers. This is possibly attributable, in part, to older drivers’ lowered ability to perceive and react to road hazards,^28^^,^^29^ which affects their ability to avoid conflicts with bicyclists and pedestrians. Bicyclists and pedestrians can suddenly appear and cross the road, especially in residential areas where the roads are narrow without separate sidewalks and bicycle lanes.

Nevertheless, in terms of the total number of injured persons per at-fault MVC, older drivers did not pose a high risk of injuries to those involved in their at-fault MVCs. This implies that the consequences of MVCs caused by older drivers are, on average, not more serious than those caused by other drivers. However, the risk of injuries to older drivers and their passengers was notably high in their MVCs. This is likely because older drivers are physically fragile^17^^,^^18^ and often drive with older passengers.^30^

Based on our findings, continued efforts are needed to reduce older drivers’ MVCs, especially with bicyclists and pedestrians; however, we cannot draw a conclusion that older drivers pose excessive injury risks to other road users, which would support the claims that they should be restricted from driving. We believe that, if it is possible to identify older drivers who are at high-risk of causing MVCs, they should be dissuaded from driving, but generally, older drivers should be assisted in driving safely for at least two reasons.

The first is the risk of traffic injuries when turning to vulnerable road users. In 2021, 58% of road traffic deaths occurred among people aged ≥65 years, 49% of road traffic deaths occurred among pedestrians and bicyclists, and 75% of pedestrian and bicyclist deaths occurred among people aged ≥65 years.^31^ The proportion of older adults and vulnerable road users in traffic deaths in Japan is two to three times as high as that in other high-income countries.^13^ The other reason is the adverse effects of driving cessation on social life and health, as suggested earlier.^8^^–^^11^

This study has some limitations. First, using aggregated data, we could not identify serious MVCs in terms of the number of injured persons in one MVC (ie, high-profile MVCs resulting in multiple victims). Thus, we only showed the average number of injured persons per MVC. We are unsure whether older drivers caused high-profile MVCs. Second, using the number of at-fault MVCs per licensed driver, we might have underestimated older drivers’ risk of at-fault MVCs relative to their driving exposure (per distance driven) because the distance driven by older drivers is less than that driven by drivers in other age groups. However, the trend of at-fault MVC risk across the sex and age groups of drivers identified in this study is generally the same as that reported in our previous study using the number of at-fault MVCs per distance driven^14^ and in similar studies in several other countries.^15^^–^^21^

In conclusion, the risk of at-fault MVCs increased as at-fault drivers advanced in age after middle age. However, the risk of at-fault MVCs among older drivers did not exceed that among young drivers. Moreover, older drivers did not pose a high risk of injuries to their collided counterparts; however, they posed a high risk to themselves and their passengers in their at-fault MVCs.
